# Antibacterial and antibiofilm effects of essential oil components, EDTA and HLE disinfectant solution on *Enterococcus*, *Pseudomonas* and *Staphylococcus* sp. multiresistant strains isolated along the meat production chain

**DOI:** 10.3389/fmicb.2022.1014169

**Published:** 2022-10-10

**Authors:** Natacha Caballero Gómez, Julia Manetsberger, Nabil Benomar, Sonia Castillo Gutiérrez, Hikmate Abriouel

**Affiliations:** ^1^Área de Microbiología, Departamento de Ciencias de la Salud, Facultad de Ciencias Experimentales, Universidad de Jaén, Jaén, Spain; ^2^Área de Estadística e Investigación Operativa, Departamento de Estadística e Investigación Operativa, Facultad de Ciencias Experimentales, Universidad de Jaén, Jaén, Spain

**Keywords:** multidrug resistant bacteria, antibiotics, resistance genes, essential oil components, HLE disinfectant solution, EDTA, synergy, biofilm

## Abstract

The spread of multidrug resistant (MDR) bacteria and resistance genes along the food chain and the environment has become a global, but silent pandemic. To face this challenge, it is of outmost importance to develop efficient strategies to reduce potential contamination by these agents. In the present study, 30 strains of *Enterococcus* sp., *Staphylococcus* sp. and *Pseudomonas* sp. isolated from various surfaces throughout the meat production chain in a goat and lamb slaughterhouse were characterized as MDR bacteria harboring several antibiotic resistance genes (ARGs). The antimicrobial efficacy of natural essential oil components “EOCs” (carvacrol “CA,” cinnamaldehyde “CIN,” eugenol “EU,” geraniol “GE,” limonene “LI” and thymol “TH”), HLE disinfectant solution (3–6% H_2_O_2_; 2.2–4.4% lactic acid and 12.5–25 mM EDTA in water) and EDTA was tested against these MDR bacteria. Results showed that Minimum Inhibitory Concentrations (MIC) were compound and strain dependent. In addition, the synergistic effect of these antimicrobials was evaluated at 1/2 MIC. Here our study showed particularly promising results regarding the inhibitory effect at sub-inhibitory concentrations, which were confirmed by the analysis of bacterial growth dynamics over 72 h. Furthermore, the inhibitory effect of EOCs, HLE disinfectant solution and EDTA or their combinations was studied in developing and established biofilms of MDR bacteria obtaining variable results depending on the morphological structure of the tested strain and the phenolic character of the EOCs. Importantly, the combination of EOCs with HLE or EDTA showed particularly positive results given the effective inhibition of biofilm formation. Moreover, the synergistic combinations of EU and HLE/EDTA, TH, CA, GE, LI or CIN + EDTA/HLE caused log reductions in established biofilms of several strains (1–6 log_10_ CFU) depending on the species and the combination used, with *Pseudomonas* sp. strains being the most susceptible. Given these results, we propose novel antimicrobial formulations based on the combination of sub-inhibitory concentrations of EOCs and HLE or EDTA as a highly promising alternative to currently used approaches. This novel strategy notably shows great potential to efficiently decrease the emergence and spread of MDR bacteria and ARGs in the food chain and the environment, thus supporting the decrease of resistomes and pathogenesis in clinical and industrial areas while preserving the antibiotic therapeutic action.

## Introduction

The spread of multidrug resistant (MDR) bacteria and related resistance genes in the food chain and the environment is considered a silent pandemic, presenting a severe global challenge. This pandemic is to a large extend caused by the extensive use and abuse of antibiotics in veterinary and clinical therapy as well as agriculture for decades. This has notably caused and increased the emergence of new MDR bacteria and promoted the prevalence of resistance genes, especially those acquired by horizontal gene transfer into the human and animal microbiomes (Laxminarayan, 2014). Antibiotic resistant bacteria (ARB) and their antibiotic resistance genes (ARGs) can spread to humans throughout the food chain *via* contaminated animals, meat products and other foods, or directly through the environment (i.e., air, water, and soil) (Founou et al., 2016). The infections caused by pathogens that carry ARGs might result in high morbidity and mortality and thus in turn result in increased health care costs. Several studies have demonstrated that the prevalence of these ARB is increasing rapidly across the globe (Subramaniam and Girish, 2020) causing the loss of antibiotic efficacy. If not addressed in time, this could potentially lead to the regression into the pre-antibiotic era (Zheng et al., 2021), with no antibiotics available for even the simplest treatment of microbial infections. As mentioned above, animal products destined for human consumption are considered one of the main reservoirs of ARB and ARGs, reinforcing the importance of controlling zoonotic pathogens in meat and other animal products through a complete and continuous farm to fork examination (Lavilla Lerma et al., 2013, 2014a,2014b,^2015^; Campos Calero et al., 2018).

To address the severe challenges caused by the global ARB and ARGs spread, the use of antibacterial strategies in the food chain is crucial. In this sense, several antibacterial agents have been proposed, however, their efficacy, safety and sustainability are of major concern in antibacterial formulations destined for the use in the food chain. EDTA is a metal-chelating agent of divalent cations commonly used in food preservation applications and previous studies demonstrated its antimicrobial-enhancing properties. In particular, EDTA has an inhibitory effect on multidrug efflux pumps in *Escherichia coli, Pseudomonas aeruginosa and Enterococcus sp.* By decreasing gene expression, thus allowing other antimicrobial agents to accumulate in the bacterial cells and resulting in greater impairment of cellular functions (Chaudhary and Payasi, 2012; Chaudhary et al., 2012; Lavilla Lerma et al., 2014c). On the other hand, the new HLE disinfectant solution- composed of hydrogen peroxide, lactic acid and EDTA- which was recently developed by our group—showed promising antimicrobial and antibiofilm activity, efficiently destabilizing and eliminating preformed biofilms (Abriouel et al., 2018). Furthermore, sub-inhibitory concentrations of this disinfectant inhibited the expression of multidrug EfrAB, NorE and MexCD efflux pumps, which could represent a promising alternative to limiting and impeding the spread of MDR bacteria in the food chain and the environment. This could consequently result in minimizing the selective pressure by the use of systemic antibiotics and disinfectants (Abriouel et al., 2018).

Recently, pure compounds from natural products (e.g., EOCs) are gaining importance as potentially promising complementary and alternative medicines for the treatment of various diseases. Notably, it was shown that single natural compounds such as curcumin and resveratrol could target DNA, mRNA, protein, and even micro-RNA (Lin et al., 2017). In particular, over the last decades, the beneficial properties of EOCs from plant extracts have been demonstrated. For example, geraniol -a pure botanical compound without adverse effects- has been identified as promising novel drug candidate for various diseases, notably due to its ability to regulate protein expression (Lei et al., 2019). The monoterpenes 2-isopropyl-5-methylphenol and 5-isopropyl-2-methylphenol, also known as thymol and carvacrol, have also received great attention in recent years due to their antimicrobial and health-beneficial properties (Kachur and Suntres, 2020; Alagawany et al., 2021). They are naturally present in a large number of plants including oregano, thyme, sweet basil, black cumin and savory (Salehi et al., 2018; Günes-Bayir et al., 2019) and plants with high levels of both compounds have been used for centuries in traditional medicine in many parts of the world (Han et al., 2017; Miara et al., 2019). However, more studies are needed to better understand their bioactive properties, including antimicrobial, antioxidant and antiviral activity, as well as their health-promoting properties (Sharifi-Rad et al., 2018; Diniz do Nascimento et al., 2020; Rathod et al., 2021). Furthermore, eugenol (an integral component of clove oil) has been authorized as food preservatives in various countries including China, the United States of America and the European Union. Many oral care products containing clove oil have also been commercialized in China, stating health benefits against halitosis, dental plaque, oral bacteria, and allergic tooth pain (Zhang et al., 2009). However, the effective concentration and antibacterial spectrum of this potential preservative is still not very clear, limiting its application in the food industry (Cortés-Rojas et al., 2014; Hu et al., 2018). Considering the above, the current study aims to develop new strategies to fight multiresistant pathogens and limit their spread along the food chain. The study notably took advantage of a collection of *Enterococcus* sp., *Pseudomonas* sp. And *Staphylococcus* sp. Strains, previously isolated from slaughterhouse surfaces throughout meat production (Lavilla Lerma et al., 2013, 2014b). Firstly, phenotypical and genotypical antibiotic resistance were evaluated with the objective to determine their multiresistant profile. Then, the application of different antimicrobials treatment strategies individually or in synergy using chelating agents, the novel HLE disinfectant solution and the promising natural active EOCs against planktonic and sessile bacteria were developed and evaluated with the aim to potentiate their antimicrobial activity, while ensuring their safety and sustainability.

## Materials and methods

### Bacterial strains and growth conditions

The study was conducted with *Enterococcus* sp. (10 strains), *Pseudomonas* sp. (10 strains) and *Staphylococcus* sp. (10 strains) strains (Table 1) isolated from different surfaces (entrance, sacrifice room, fridges, cutting room and white room) of a local goat and lamb slaughterhouse in Jaén (Spain) as described previously by Lavilla Lerma et al. (2013). All strains were maintained and stored in Tryptone Soy Broth (TSB; Scharlab, Barcelona, Spain) containing 20% glycerol at −80°C. *Enterococcus* sp. And *Staphylococcus* sp. Strains were cultivated in TSB at 37°C, while *Pseudomonas* sp. Strains were cultivated in TSB at 25°C.

**TABLE 1:** Bacterial strains used in this study.

*Enterococcus* sp.*		*Staphylococcus* sp.*		*Pseudomonas* sp.*	
Strain	Source	Strain	Source	Strain	Source
M17M10	Cutting room	M3G13	Sacrifice room	1T08	Entrance
M13M11	Cutting room	M7G10	Sacrifice room	M13K11	Cutting room
M18M10.2	Cutting room	M7G11	Sacrifice room	M15K10	Cutting room
M18M11	Cutting room	M9G8	Sacrifice room	M22K12	Fridge 3
M27M07	Fridge 4	M9G13	Sacrifice room	M24T02	Fridge 3
M28M08	Fridge 4	M9G11	Sacrifice room	M24T02.2	Fridge 3
M28M01	Fridge 4	M19G13	Cutting room	M24T11.2	Fridge 3
M28M11	Fridge 4	M27G07	Fridge 4	M32K04	White room
M28M12	Fridge 4	M30G12	White room	M33K08	White room
M30M13	White room	M31G11	White room	M33T02.2	White room

*Strains were isolated throughout meat chain production in a lamb and goat slaughterhouse of Jaén (Lavilla Lerma et al., 2013).

### Phenotypic and genotypic antibiotic resistance of pathogenic bacteria

#### Phenotypic antibiotic susceptibility

Antimicrobial susceptibility was determined using the disk diffusion method performed on Mueller-Hinton Agar (Merck). The following eleven antibiotics were tested: ampicillin 10 μg (AMP), amoxicillin-clavulanic acid 30 μg (AMC), gentamicin 10 μg (CN), ciprofloxacin 5 μg (CIP), tetracycline 30 μg (TE), rifampicin 5 μg (RD), erythromycin 15 μg (E), nitrofurantoin 300 μg (F), chloramphenicol 30 μg (CL), imipenem 10 μg (IPM) and cefuroxime 30 μg (CX). Cartridges with commercially prepared paper disks containing the appropriate antibiotic dosage were purchased from Oxoid (United Kingdom).

An overnight culture of isolates was streaked evenly on Mueller-Hinton agar plates and the antibiotic disks were placed on the surface. Zone diameters were recorded after overnight incubation at 37°C and the strains were classified as resistant and susceptible according mainly to the criteria from Clinical and Laboratory Standards Institute [CLSI] (2020). In other cases, EUCAST (2022) and CASFM/EUCAST Société Française de Microbiologie (2019) were used as follows: EUCAST (2022) for imipenem and gentamycin in the case of *Enterococcus* sp., rifampicin, erythromycin, cefuroxime and gentamycin for *Staphylococcus* sp.; and CASFM/EUCAST Société Française de Microbiologie (2019) was used to evaluate resistance to rifampicin in *Enterococcus* sp.

#### Molecular screening of resistance determinants

Total DNA extractions were done using the Zymo BIOMICS DNA Miniprep Kit (Zymo Research, California, United States) according to the manufacturer’s instructions. DNA quantification and quality assessment were done with a NanoDrop 2000 spectrophotometer (Thermo Scientific). PCR amplifications of well-known structural genes associated with resistance to beta-lactams (*bla*OXA, *bla*CTXa-CTXb, *bla*SHV-1 and *bla*TEM), chloramphenicol (*catA1*, *catA2*, *catA3* and *catB3*), macrolides (*ereA*, *ereB*, *ermA*, *ermB*, *msrA* and *mrsB*, and *mefA*), tetracycline (*tetA*, *tetB*, *tetO* and *tetQ*), aminoglycosides [*aad(E)*, *aphA-3*, *aac(6* = *)-Ie-aph(2* = *)-Ia*, *aph(2* = *)-Ib*, *aph(2* = *)-Ic*, *aph(2* = *)- Id*, *aph(3* = *)-IIIa* and *ant(4* = *)-Ia*], trimethoprim (*dfrA* and *dfrD*) and sulphonamide (*sulI*, *sulII* and *sulIII*) were performed following the methods described in Table 2. Efflux pumps mediating multiple antibiotic resistance also were included in this study such as AcrA, AcrB, TolC, MexAB, MexCD and MexXY (Table 2).

**TABLE 2:** Primers and conditions used in this study.

Target	Primer	Sequence (5′–3′)	Annealing temperature (°C)	References
*bla*_*CTX–A*_	blaCTX-A F	CGGGCRATGGCGCARAC	60	Roschanski et al., 2014
	blaCTX-A R	GCRCCGGTSGTATTGCC		
*bla*_*CTX–B*_	blaCTX-B F	ACCGAGCCSACGCTCAA	60	Roschanski et al., 2014
	blaCTX-B R	CCGCTGCCGGTTTTATC		
*bla*_*OXA*_	blaOXA-F	ACCAGATTCAACTTTCAA	50	Birkett et al., 2007
	blaOXA-R	TCTTGGCTTTTATGCTTG		
*bla*_*TEM*_	blaTEM-F	TCGGGGAAATGTGCG	55	Kim et al., 2013
	bla TEM-R	GGAATAAGGGCGACA		
*bla*_*SHV–1*_	blaSHV-F	TGATTTATCTGCGGGATACG	55	Knapp et al., 2010
	blaSHV-R	TTAGCGTTGCCAGTGCTCG		
*tet(A)*	tetA-F	GCTACATCCTGCTTGCCTTC	55	Knapp et al., 2010
	tetA-R	CATAGATCG CCG TGAAGAGG		
*tet(B)*	tetB-F	TTGGTTAGGGGCAAGTTTTG	55	Ng et al., 2001
	tetB-R	GTAATGGGCCAATAACACCG		
*tet(O)*	tetO-F	AACTTAGGCATTCTGGCTCAC	55	Ng et al., 2001
	tetO-R	TCCCACTGTTCCATATCGTCA		
*tet(Q)*	tetQ-F	TTATACTTCCTCCGGCATCG	55	Ng et al., 2001
	tetQ-R	ATCGGTTCGAGAATGTCCAC		
*catA1*	catA1-F	CGCCTGATGAATGCTCATCCG	58	Ng et al., 2001
	catA1-R	CCTGCCACTCATCGCAGTAC		
*catA2*	catA2-F	ATGAATTTTACCAGAATTGATCTGAA	58	Kim et al., 2013
	catA2-R	ATTTCAGTATGTTATCACACATCA		
*catA3*	catA3-F	AAATTGGGTTCGCCGTGA	58	Kim et al., 2013
	catA3-R	ATTTACTGTTACACAACT CTTGTA		
*catB3*	catB3-F	TCAAAGGCAAGCTGCTTTCTGAGC	58	Kim et al., 2013
	catB3-R	TATTAGACGAGCACAGCATGGGCA		
*ermA*	ermA1	TCTAAAAAGCATGTAAAAGAA	52	Kim et al., 2013
	ermA2	CTTCGATAGTTTATTTAATATTAGT		
*ermB*	ermB1	GAAAAGGTACTCAACCAAATA	52	Sutcliffe et al., 1996
	ermB2	AGTAACGGTACTTAAATTGTTTAC		
*mefA*	mefA-F	AGTATCATTAATCACTAGTGC	55	Sutcliffe et al., 1996
	mefA-R	TTCTTCTGGTACTAAAAG TGG		
*ereA*	EreA-F	AACACCCTGAACCCAAGGGACG	55	Sutcliffe et al., 1996
	EreA-R	CTTCACATCCGGATTCGCTCGA		
*ereB*	EreB-F	CATATAATCATCACCAATGGCA	55	Sutcliffe et al., 1996
	EreB-R	AGAAATGGAGGTTCATACTTACCA		
*msrA*	msrA-F	GGCACAATAAGAGTGTTTAAAGG	50	Sutcliffe et al., 1996
	msrA-R	AAGTTATATCATGAATAGATTGTCCTGTT		
*msrB*	msrB-F	TATGATATCCATAATAATTATCCAATC	50	Lina et al., 1999
	msrB-R	AAGTTATATCATGAATAGATTGTCCTGTT		
*aadE*	aadEI	GCAGAACAGGATGAACGTATTCG	55	Lina et al., 1999
	aadEII	ATCAGTCGGAACTATGTCCC		
*aphA3*	aphA3-F	GGGACCACCTATGATGTGGAACG	58	Klare et al., 2007
	aphA3-R	CAGGCTTGATCCCCAGTAAGTC		
*aac(6′)Ie*-	aac(6′)-Ie-aph(2′′)-Ia-F	CAGAGCCTTGGGAAGATGAAG	55	Kim et al., 2013
*aph(2′′)-Ia*	aac(6′)-Ie-aph(2′′)-Ia-R	CCTCGTGTAATTCATGTTCTGGC		
*aph(2′′)-Ib*	aph(2′′)-Ib-F	CTTGGACGCTGAGATATATGAGCAC	55	Vakulenko et al., 2003
	aph(2′′)-Ib-R	GTTTGTAGCAATTCAGAAACACCCTT		
*aph(2′′)-Ic*	aph(2′′)-Ic-F	CCACAATGATAATGACTCAGTTCCC	55	Vakulenko et al., 2003
	aph(2′′)-Ic-R	CCACAGCTTCCGATAGCAAGAG		
*aph(2′′)-Id*	aph(2′′)-Id-F	GTGGTTTTTACAGGAATGCCATC	55	Vakulenko et al., 2003
	aph(2′′)-Id-R	CCCTCTTCATACCAATCCATATAACC		
*aph(3′)-III*	aph(3′)-IIIa-F	GGCTAAAATGAGAATATCACCGG	55	Vakulenko et al., 2003
	aph(3′)-IIIa-R	CTTTAAAAAATCATACAGCTCGCG		
ant(4′)-Ia	ant(4′)-Ia-F	CAAACTGCTAAATCGGTAGAAGCC	55	Vakulenko et al., 2003
	ant(4’)-Ia-R	GGAAAGTTGACCAGACATTACGAACT		
*sulI*	sulI-F	CGCACCGGAAACATCGCTGCAC	56	Vakulenko et al., 2003
	sulI-R	TGAAGTTCCGCCGCAAGGCTCG		
*sulII*	sulII-F	TCCGGTGGAGGCCGGTATCTG G	61	Pei et al., 2006
	sulII-R	CGGGAATGCCATCTGCCTTGAG		
*sulIII*	sulIII-F	TCCGTTCAGCGAATTGGTGCAG	60	Pei et al., 2006
	sulIII-R	TTCGTTCACGCCTTACACCAGC		
*dfrA*	dfrA1	CTTTTCTACGCACTAAATGTAAG	50	Liu et al., 2009
	dfrA2	CATTATCAATAATTGTCGCTCAC		
*dfrD*	drfD1	GGAAGGGCTTTACCTGACAGAAG	50	Liu et al., 2009
	dfrD2	CGACATAAGGCAAGAACATAACATA		
*acrA*	acrA-F	CTCTCAGGCAGCTTAGCCCTAA	60	Swick et al., 2011
	acrA-R	TGCAGAGGTTCAGTTTTGACTGTT		
*acrB*	acrB-F	GGTCGATTCCGTTCTCCGTTA	60	Swick et al., 2011
	acrB-R	CTACCTGGAAGTAAACGTCATTGGT		
*tolC*	tolC-F	AAGCCGAAAAACGCAACCT	57	Swick et al., 2011
	tolC-R	CAGAGTCGGTAAGTGACCATC		
*mexB*	MxB- U	CAAGGGCGTCGGTGACTTCCAG	62	Oh et al., 2003
	MxB- L	ACCTGGGAACCGTCGGGATTGA		
*mexY*	MxY- U	GGACCACGCCGAAACCGAACG	62	Oh et al., 2003
	MxY- L	CGCCGCAACTGACCCGCTACA		
*mexD*	MxD- U	GGAGTTCGGCCAGGTAGTGCTG	62	Oh et al., 2003
	MxD- L	ACTGCATGTCCTCGGGGAAGAA		

## Antibacterial assays

### Antimicrobial agents

HLE disinfectant solution (3–6% H_2_O_2_; 2.2–4.4% lactic acid and 12.5–25 mM EDTA in water) (HLE components were obtained from Sigma-Aldrich, Spain) prepared as described by Abriouel et al. (2018) and ethylenediamine tetra acetic acid (EDTA) (Sigma-Aldrich, Spain) prepared in MQ sterile water were tested. EOCs used in this study were: geraniol (GE), carvacrol (CA), eugenol (EU), limonene (LI), thymol (TH) and *trans*-Cinnamaldehyde (CIN), obtained from Sigma-Aldrich (Spain).

### Minimum inhibitory concentration determination

The broth micro-dilution method was used to determine the minimum inhibitory concentration (MIC) of HLE, EDTA and EOCs (CA, CIN, EU, GE, LI and TH), as previously described (Clinical and Laboratory Standards Institute [CLSI], 2020). Overnight bacterial cultures grown at 37°C for 24 h, were diluted 1/10 (v/v) in fresh Mueller Hinton (MH) broth or Mueller Hinton II broth-Cation adjusted (MHII) in the case of *Pseudomonas* corresponding to an inoculum density of 0.5 McFarland, and 20 μl were added to each well of a 96-well microtiter plate. Then, 180 μl of MH or MHII broth supplemented with different concentrations of antimicrobials were added prior to incubation for 24 h under aerobic conditions. The ranges tested for each antimicrobial depended greatly on the antimicrobial agent tested: 0.0031–25% for HLE (v/v); 0.0001–200 mM for EDTA and 10–450 μg/ml for EOCs (CA, CIN, EU, GE, LI and TH).

Bacterial growth was evaluated by the presence of turbidity and MIC was defined as the lowest concentration of the antimicrobial agent that inhibited visible growth. Each experiment was performed in triplicate.

### Evaluation of synergy between EOCs and HLE or EDTA

The synergistic effects between different potential antimicrobial compounds (EOCs and HLE or EDTA) were evaluated as described above. Overnight bacterial cultures were diluted 1/10 (v/v) in fresh MH or MHII broth and the bacterial suspension was adjusted to match the turbidity standard of 0.5 McFarland units. Synergistic effects were tested using the “Multiple-Combination Bacterial Test” (MCBT) as described by Doern (2014). 20 μl of overnight bacterial culture (adjusted at 0.5 McFarland units), a quantity of antimicrobials (EDTA, HLE or EOCs) representing 1/2 of its MIC, and a quantity of MH or MHII broth to obtain a final volume of 200 μl. After overnight incubation the synergistic effects were evaluated by turbidity inspection. Each experiment was done in triplicate.

### Determination of growth kinetic of pathogenic bacteria and the effect of antimicrobials

Bacterial growth kinetic in the presence of antimicrobials and their combinations was determined as described in the previous sections (MIC and MCBT). Three strains representative of each species were selected (*Enterococcus* sp. strains M13M11, M28M11 and M28M12; *Staphylococcus* sp. strains M7G10, M9G8 and M31G11; and *Pseudomonas* sp. strains M13K11, M22K12 and M33T02.2), considering all the potential synergistic effects obtained with EOCs (CA, CIN, EU, GE, LI and TH) and EDTA or HLE. 96-well microtiter plates were incubated at 37°C (*Enterococcus* sp. or *Staphylococcus* sp.) or 25°C (*Pseudomonas* sp.) ± 0.3°C and analyzed over a period of 72 h. The optical density (OD) at 580 nm was determined for each well using a Tecan Infinite M200 multimode microplate reader equipped with monochromatoroptics (Tecan Group Ltd., Männedorf, Switzerland). Before each measurement, orbital shaking conditions were selected (4 mm amplitude and 15 s shaking cycles), and measurements were taken every 60 min using the multiple-reads-per-well mode (filled-circle alignment, 3 × 3 spots, five reads per well, border 2,000 μm). Each experiment was performed in triplicate.

### Quantification of biofilm formation of pathogenic bacteria

The quantification of biofilm production of selected strains (*Enterococcus* sp. strains M13M11, M28M11 and M28M12; *Staphylococcus* sp. strains M7G10, M31G11 and M9G8; and *Pseudomonas* sp. strains M13K11, M22K12 and M33T02.2) in light of synergistic effects previously detected, was performed as described by Caballero Gómez et al. (2016). The wells of a sterile 12-well polystyrene microtiter plate (TPP, Switzerland) were filled with 2 ml of TSB broth. Overnight bacterial cultures were diluted 1/10 (v/v) in fresh TSB and the bacterial suspension was adjusted to match the turbidity standard of 0.5 McFarland units, then 200 μl were added to each well. The plates were incubated aerobically for 24 h at the strain appropriate temperature. To quantify the biofilm formation, the wells were gently washed three times with 2 ml of Phosphate Buffer Saline (PBS). The adhered bacteria were fixed with 2 ml methanol (Panreac) for 15 min, and then the microplates were emptied and dried at room temperature. Subsequently, 2 ml of a 2% (v/v) crystal violet solution was added to each well and kept at room temperature for 5 min. Excess stain was removed under gently running tap water and stain was released from adherent cells with 2 ml of 33% (v/v) glacial acetic acid. The optical density (OD) of each well was measured at 620 nm using a plate reader (Microplate Tecan). Non-inoculated TSB was used as negative control and the cut-off value (OD_*C*_) was defined as the mean OD value of the negative control. Strains were classified according to Borges et al. (2012) based on the measured OD as follows: non-biofilm producers (OD ≤ OD_*C*_), weak (OD_*C*_ < OD ≤ 2 × ODC), moderate (2 × OD_*C*_ < OD ≤ 4 × OD_*C*_) or strong biofilm producers (4 × OD_*C*_ < OD). Each experiment was done in triplicate.

## Evaluation of anti-biofilm efficacy of antimicrobial agents against pathogenic bacteria

### Effect of antimicrobial agents on biofilm formation

The antibiofilm properties of antimicrobial agents (EDTA, HLE or EOCs) were determined for strains (*Enterococcus* sp. strains M13M11, M28M11 and M28M12; *Staphylococcus* sp. strains M7G10, M31G11 and M9G8; and *Pseudomonas* sp. strains M13K11, M22K12 and M33T02.2) selected based on the synergistic effects as previously detected. 20 μl of 1/10 (v/v) diluted overnight bacterial cultures, adjusted to match the turbidity standard of 0.5 McFarland units, were distributed in a microtiter plate and 180 μl of TSB supplemented with MIC and 1/2 MIC concentrations for different antimicrobial agents and their combination were added, respectively. Controls without antimicrobials consisting solely of 180 μl of TSB broth were used. Plates were incubated at 37°C (*Enterococcus* sp. and *Staphylococcus* sp.) or at 25°C (*Pseudomonas* sp.) under aerobic conditions for 24 h. Quantification of biofilms was performed as described above. The percentage of inhibition of biofilm formation was determined using the following formula as described by Zmantar et al. (2017). Each experiment was performed in triplicate.


[O⁢D⁢g⁢r⁢o⁢w⁢t⁢h⁢c⁢o⁢n⁢t⁢r⁢o⁢l-O⁢D⁢s⁢a⁢m⁢p⁢l⁢eO⁢D⁢g⁢r⁢o⁢w⁢t⁢h⁢c⁢o⁢n⁢t⁢r⁢o⁢l]⁢x⁢ 100

### Effect of antimicrobial agents on preformed biofilms of pathogenic bacteria

Biofilms of the selected multiresistant pathogenic bacteria (*Enterococcus* sp. strains M13M11, M28M11 and M28M12; *Staphylococcus* sp. strains M7G10, M31G11 and M9G8; and *Pseudomonas* sp. strains M13K11, M22K12 and M33T02.2) were prepared as described above. Non-adhered bacteria were eliminated after 24 h incubation and the resulting biofilms were washed with sterile PBS twice and then treated with MIC of antimicrobial agents (determined previously) as well as with their combination, for 30 min at room temperature. After treatment, compounds were removed and the wells were washed with 200 μl of D/E Neutralizing broth (Difco, Spain) for 5 min at room temperature and then washed with 200 μl of PBS. The obtained suspensions of preformed biofilms (treated or not with antimicrobial agents) were transferred into sterile tubes and vortexed for 30 s. Serial dilutions were prepared in 0.85% (w/v) saline solution and then plated on TSA. The plates were incubated at 37°C (*Enterococcus* sp. or *Staphylococcus* sp.) or 25°C (*Pseudomonas* sp.) for 24 h to determine bacterial counts (CFU/ml) and thus log reduction after each treatment. Each experiment was performed in triplicate.

### Statistical analysis

Statistical analyses were conducted using Excel 2016 (Microsoft Corporation, Redmond, WA, United States) to determine averages and standard deviations. All analyses were performed in triplicate. Synergy data were analyzed by Student’s *t* test using Excel 2016. Statistical calculations were based on a confidence level ≥ 95% (*P* < 0.05) which was considered statistically significant.

## Results

### Phenotypic and genotypic antibiotic resistance profiles of pathogenic bacteria

Enterococci were phenotypically resistant to cefuroxime CXM30 and rifampicin RD5 (100%), ciprofloxacin CIP5 and nitrofurantoin F300 (80%), tetracycline TE30 (70%), chloramphenicol CE30 (50%), imipenem IPM10 (40%), amoxicillin-clavulanic acid AMC30 (40%) and 20% of strains were resistant to ampicillin AMP10, erythromycin E15 and gentamicin CN10 (Table 3). Almost all strains harbored macrolide resistance determinants (90%), while efflux pump genes, aminoglycoside and chloramphenicol determinants were presents in 70% and 60% of strains, respectively. However, trimethoprim and sulfonamide resistance genes were only present in 10% of enterococci and no resistance determinants were detected for tetracyclines and beta-lactams (Table 3).

**TABLE 3:** Phenotypic and genotypic antibiotic resistance profile of bacterial strains isolated throughout meat chain production in a lamb and goat slaughterhouse of Jaén.

Bacterial strains	Phenotypic resistance profile	Genotypic resistance determinants
***Enterococcus* sp.**		
M27M07	CIP5, RD5, CXM30, F300, IPM10	*catA3*, *mexD*, *sulI*
M28M08	CIP5, RD5, CXM30, F300, AMC30	*mexB*, *aac(6* = *)-Ie-aph(2* = *)-Ia*, *ermB*
M28M11	CE30, AMP10, CIP5, RD5, CXM30, CN10, F300, IPM10, AMC30	*catA3*, *ermA*, *mexB*
M18M11	CE30, TE30, CIP5, RD5, CXM30, F300	*catA3*, *ermA*, *ereA*, *aphA-3*
M30M13	TE30, CIP5, RD5, CXM30	*ereA*, *ereB*, *aac(6* = *)-Ie-aph(2* = *)-Ia*, *dfrA*
M17M10	TE30, AMP10, CIP5, RD5, CXM30, F300, IPM10, AMC30	*tolC*, *ereB*, *ant(4* = *)-Ia*, *aac(6* = *)-Ie-aph(2* = *)-Ia*
M18M10.2	CE30, TE30, CIP5, RD5, E15, CXM30, F300	*catA3*, *ermA*, *aphA-3*
M13M11	CE30, TE30, RD5, CXM30, F300, AMC30	*catA3*, *ermA*, *mexB*
M28M12	CE30, CIP5, RD5, CXM30, CN10, F300, IPM10, TE30	*mexY*, *ereB*, *aac(6* = *)-Ie-aph(2* = *)-Ia aad(E)*, *ant(4* = *)-Ia*
M28M01	TE30, RD5, CXM30, E15	*catB3*, *msrB*, *tolC*, *mexD*
***Staphylococcus* sp.**		
M9G13	RD5, E15, CN10, TE30, CE30	*bla*_*SHV–1*_, *catA3*, *aad(E)*, *mexD*
M19G13	CIP5, RD5, E15, CN10, TE30, CE30	*aad(E)*, *ermB*, *catA3*, *bla*_*SHV–1*_, *mexD*
M9G11	RD5, E15, CN10, CIP5, TE30, CE30	*bla*_*SHV–1*_, *bla*_*TEM*_, *tetB*, *ermB*, *aad(E)*, *tolC*, *mexD*
M31G11	RD5, E15, CN10, CIP5, TE30, CE30, F300	*tolC*, *mexD*, *mexY*, *ermB*, *catA1*, *catA3*, *bla*_*TEM*_
M7G10	RD5, E15, CN10, CIP5, TE30, CXM30	*ermB*, *mexD*, *ereA*, catA3, *bla*_*CTX–A*_
M30G12	RD5, E15, CIP5, TE30, CE30	*catA3*, *ermB*, *aad(E)*, *mexY*
M3G13	E15, TE30, CE30, CXM30	*aad(E)*, *ermB*, *catA3*
M7G11	RD5, CN10, CIP5, E15, TE30, CXM30	*catA3*, *aad(E)*, *sulI*, *mexY*
M9G8	RD5, E15, CN10, E15, TE30, CXM30, CE30	*catB3*, *bla*_*TEM*_, *aphA-3*
M27G07	RD5, CIP5, CN10, E15, TE30, CXM30, CE30	*aad(E)*, *mexY*, *ereB*
***Pseudomonas* sp.**		
M33T02.2	AMP10, CXM30, RD5, CIP5, F300	*bla_*SHV–1*_, ermB, aad(E), tolC*
M22K12	AMP10, CXM30, CIP5, IPM10, CN10, CE30	*tolC, ermB, catA1, catA3, bla_*SHV–1*_, bla_*CTX–B*_*
M15K10	AMP10, CXM30, RD5, CIP5, CE30, F300, AMC30	*bla_*SHV–1*_, bla_*TEM*_, catA1, catA3, catB3, aad(E), tolC, mexB*
M13K11	CXM30, CIP5, CE30, TE30	*tolC, mexB, sulI, aad(E), ermB, catA3, bla_*SHV–1*_, bla_*CTX–B*_*
M24T11.2	AMP10, CXM30, RD5, CIP5, CE30, F300, AMC30, IPM10	*bla_*SHV–1*_, catA3, ermB, aad(E), sulI, tolC, mexB*
1T08	AMP10, CXM30, CIP5, CE30, F300, E15	*sulI, tolC, mexB, catA3, ermB, aad(E), bla_*CTX–B*_*
M32K04	AMP10, CXM30, RD5, CIP5, CE30, F300, IPM10	*bla_*TEM*_, catB3, ereA, sulI, tolC, mexB*
M24T02.2	AMP10, CXM30, CIP5, CE30, F300, AMC30, RD5	*tolC, mexB, bla_*TEM*_, ereA, ereB*
M33K08	AMP10, CXM30, CIP5, CE30, F300, RD5	*catA3*, *catB3*, *ermB*, *aad(E)*, *tolC*
M24T02	AMP10, CXM30, RD5, CIP5, CE30, F300, AMC30	*tolC*, *ereB*, *bla*_*TEM*_

Generally, phenotypic antibiotic resistance in *Staphylococcus* sp. strains was more homogeneous than in enterococci. All strains were resistant to tetracycline TE30 and erythromycin E15, and 90% to rifampicin RD5. Resistance to gentamicin CN10, chloramphenicol CE30 and ciprofloxacin CIP5 was detected in 70–80% of strains (Table 3). On the other hand, 50% and 10% of strains exhibited resistance to cefuroxime CXM30 and nitrofurantoin F300, respectively. However, neither of *Staphylococcus* sp. strains showed phenotypical resistance to ampicillin AMP10, amoxicillin-clavulanic acid AMC30 and imipenem IMP10. With respect to genotypic antibiotic resistance, 80% of strains showed chloramphenicol and aminoglycoside resistance genes as well as efflux pump determinants, 70% of resistance genes against macrolides and 60% against beta-lactams. Only 10% of *Staphylococcus* sp. strains exhibited sulfonamide resistance determinants and no strain showed trimethroprim resistance genes (Table 3).

Regarding *Pseudomonas* sp., phenotypic resistance was shown in 100% of strains to ciprofloxacin CIP5 and cefuroxime CXM30, 90% to ampicillin AMP10 and chloramphenicol CE30, 80% to nitrofurantoin F300, 70% to rifampicin RD5, 40% amoxicillin-clavulanic acid AMC30, 30% to imipenem IMP10 and 10% to gentamicin CN10, tetracycline TE30 and erythromycin E15 (Table 3). When antibiotic resistance genes were investigated, 100% of strains presented efflux pump determinants, 90% exhibited beta-lactam and macrolide resistance genes. However, chloramphenicol, aminoglycoside and sulfonamide resistance determinants were detected in 70%, 60% and 40% of *Pseudomonas* sp. strains, respectively. On the other hand, trimethoprim determinants were not detected (Table 3).

### Antimicrobial activity of EOCs, HLE and EDTA against pathogenic multiresistant bacteria

#### Determination of minimum inhibitory concentration of EOCs, HLE and EDTA

Cinnamaldehyde (CIN) presented the smallest MICs for all bacterial strains tested (Table 4), i.e., only low concentrations were required to drastically inhibit growth (10–50 μg/mL). However, thymol (TH), carvacrol (CA) and limonene (LI) presented MICs in the range of 100–200 μg/mL for most *Pseudomonas* sp. strains, being higher in most of cases for *Enterococcus* sp. and *Staphylococcus* sp. strains (in the range of 200–400 μg/mL) (Table 4). Regarding geraniol (GE), this compound generally showed lower MIC values for *Enterococcus* sp. (100–350 μg/mL) in comparison to *Pseudomonas* sp. (100–400 μg/mL) and *Staphylococcus* sp. (100–400 μg/mL) strains (Table 4). On the other hand, eugenol (EU) had the highest MICs for all strains (Table 4). Furthermore, as summarized in Table 4, all tested compounds exhibited antimicrobial activity against multiresistant bacterium strains, albeit depending on the compound and the tested strain.

**TABLE 4:** MIC of EOCs, HLE and EDTA against *Enterococcus* sp., *Staphylococcus* sp. and *Pseudomonas* sp. strains isolated throughout meat chain production in a lamb and goat slaughterhouse of Jaén.

Bacteria	Antimicrobials (μg/mL)							
	Thymol (TH)	Geraniol (GE)	Limonene (LI)	Carvacrol (CA)	Eugenol (EU)	Cinnamaldehyde (CIN)	HLE (%)	EDTA (mM)
***Enterococcus* sp.**								
M30M13	450	300	450	450	450	50	0.0125	20
M13M11	350	250	400	400	350	10	0.0125	2
M18M11	200	200	400	400	350	50	0.025	2
M28M11	450	100	100	100	350	50	0.025	0.0001
M27M07	300	100	250	250	450	50	0.0031	125
M18M10.2	200	250	250	250	250	50	0.0125	0.078
M28M08	200	200	150	250	200	10	0.0031	0.31
M17M10	200	250	250	250	250	10	0.0125	15.6
M28M01	350	350	350	350	350	50	0.0125	15
M28M12	350	350	350	350	350	50	0.0125	0.078
***Pseudomonas* sp.**								
M32k04	200	300	200	200	300	10	2.5	0.01
M33K08	200	250	200	200	300	10	2.5	0.1
M13K11	200	300	200	200	300	50	2.5	200
M24T02	200	400	200	200	300	10	2.5	1
M15K10	100	300	100	100	300	10	2.5	1
M22K12	300	400	300	400	400	10	2.5	0.001
M24T11.2	200	400	200	200	300	10	2.5	1
M24T02.2	200	400	200	200	300	10	2.5	1
1T08	100	100	100	100	300	10	2.5	1
M33T02.2	150	150	100	100	400	10	2.5	1
***Staphylococcus* sp.**								
M7G10	300	100	100	200	300	50	2.5	100
M9G13	300	300	300	300	300	10	0.25	10
M9G11	300	300	300	300	300	50	0.125	50
M31G11	300	300	300	300	300	50	0.025	12.5
M19G13	300	300	300	300	300	50	0.0031	6.26
M30G12	200	300	300	200	300	50	2.5	100
M3G13	300	300	300	300	300	10	0.25	100
M27G07	300	300	300	200	300	50	2.5	100
M7G11	400	400	400	400	400	10	0.0031	0.625
M9G8	300	300	300	300	300	50	25	12.5

The MICs of HLE and EDTA for the different bacterial strains used in this study are shown in Table 4. Regarding the HLE disinfectant solution, *Enterococcus* sp. strains presented high susceptibility, exhibiting lower MICs ranging from 0.0031 to 0.025% HLE (v/v). *Pseudomonas* sp. strains were less susceptible than enterococci with MICs of 2.5% HLE (v/v) for all tested strains (Table 4). Regarding *Staphylococcus* sp. strains, we observed more variability obtaining MIC values ranging from 0.0031 to 25% (v/v) depending on the strain tested (Table 4).

Concerning EDTA, *Pseudomonas* sp. strains showed the lowest MIC values ranging from 0.001 to 1 mM except for the M13K11 strain which had a MIC of 200 mM (Table 4). *Staphylococcus* sp. strains were the least susceptible to EDTA with MIC values ranging between 10 and 100 mM, while M7G11 and M19G13 strains had MIC values of 0.625 and 6.26 mM, respectively (Table 4). EDTA MICs in enterococci were strain dependent with high variability ranging between 0.0001 mM for the M28M11 strain and 125 mM for the M27M07 strain (Table 4).

#### Determination of the synergistic effect between antimicrobials against pathogenic multiresistant bacteria

The synergistic effect of EOCs and EDTA or HLE was species and strain dependent (Table 5). Nevertheless, combinations with a major number of synergies were detected for CIN + EDTA (10/10 strains), GE + HLE (9/10 strains), and LI + HLE (7/10 strains) against *Pseudomonas* sp. strains; LI + EDTA (8/10 strains), CA + EDTA (7/10 strains), and GE + EDTA (6/10 strains) against *Staphylococcus* sp. strains; and in the case of enterococci CA + EDTA showed synergistic effects against six of ten strains (Table 5). In the remaining cases, synergistic effects of EOCs and HLE or EDTA were detected against 1–5 multiresistant strains tested, with the combination of EOCs and HLE generally more effective against *Pseudomonas* sp. strains and the combination of EOCs and EDTA against Gram positive bacteria (*Enterococcus* sp. and *Staphylococcus* sp.) (Table 5).

**TABLE 5:** Synergies detected between EOCs and HLE or EDTA against *Enterococcus* sp., *Staphylococcus* sp. and *Pseudomonas* sp. strains isolated throughout meat chain production in a lamb and goat slaughterhouse of Jaén.

Bacteria	Antimicrobials											
	Thymol		Carvacrol		Limonene		Geraniol		Eugenol		Cinnamaldehyde	
	HLE	EDTA	HLE	EDTA	HLE	EDTA	HLE	EDTA	HLE	EDTA	HLE	EDTA
***Enterococcus* sp.**												
M30M13	−	**+**	−	**+**	−	**+**	−	−	−	−	**+**	−
M13M11	**+**	−	**+**	**+**	−	−	−	−	−	−	−	−
M18M11	−	−	−	−	−	−	−	−	−	−	−	−
M28M11	−	**+**	−	**+**	−	−	−	−	**+**	−	−	**+**
M27M07	−	−	−	**+**	−	**+**	−	**+**	−	**+**	−	−
M18M10.2	−	−	−	−	−	−	−	−	**+**	−	**+**	−
M28M08	−	−	−	−	−	−	−	−	−	**+**	−	**+**
M17M10	−	−	−	−	−	−	−	−	−	−	−	−
M28M01	−	−	−	**+**	−	−	−	**+**	−	−	**+**	−
M28M12	**+**	−	**+**	**+**	**+**	−	**+**	**+**	**+**	**+**	−	**+**
***Pseudomonas* sp.**												
M32K04	**+**	−	**+**	−	**+**	−	**+**	−	**+**	−	−	**+**
M33K08	−	−	**+**	−	**+**	−	**+**	−	**+**	−	−	**+**
M13K11	−	**+**	−	**+**	**+**	**+**	**+**	**+**	−	**+**	**+**	**+**
M24T02	−	−	−	−	−	**+**	**+**	−	−	−	−	**+**
M15K10	−	−	−	−	**+**	**+**	**+**	**+**	−	−	−	**+**
M22K12	−	−	**+**	−	−	−	**+**	−	−	−	−	**+**
M24T11.2	−	−	−	−	**+**	**+**	**+**	−	−	−	−	**+**
M24T02.2	−	−	−	−	**+**	**+**	**+**	**+**	−	−	−	**+**
1T08	−	−	−	−	−	−	−	−	**+**	−	−	**+**
M33T02.2	**+**	−	**+**	−	**+**	−	**+**	−	**+**	**+**	−	**+**
***Staphylococcus* sp.**												
M7G10	−	−	**+**	**+**	**+**	−	**+**	**+**	−	**+**	−	−
M9G13	−	**+**	−	−	−	**+**	−	−	−	−	−	**+**
M9G11	−	**+**	−	**+**	−	**+**	−	**+**	−	**+**	−	−
M31G11	**+**	**+**	−	**+**	−	**+**	−	−	**+**	**+**	−	−
M19G13	−	−	−	−	−	**+**	−	−	−	−	−	−
M30G12	−	−	**+**	**+**	**+**	**+**	**+**	**+**	**+**	−	−	−
M3G13	−	−	−	**+**	−	**+**	−	**+**	−	−	−	−
M27G07	−	−	−	**+**	**+**	**+**	**+**	**+**	**+**	**+**	−	−
M7G11	**+**	−	−	−	−	−	−	−	−	−	−	**+**
M9G8	−	−	−	**+**	**+**	**+**	**+**	**+**	**+**	**+**	−	**+**

+, presence of synergy; –, absence of synergy.

#### Antimicrobial effect of EOCs, HLE and EDTA on pathogenic multiresistant bacteria growth dynamics

To examine the effects of EOCs (TH, CA, EU, LI, GE and CIN) as well as their putative synergy with HLE or EDTA on the growth dynamics of specific strains -selected based on their susceptibility to all antimicrobial agents and their combinations (Table 5)- we monitored bacterial growth using an automated 96-well microtiter plate assay that allowed simultaneous cultivation and on-line analysis of bacterial growth. By examining growth over 72 h, different effective exposure times and concentrations dependent effects on the growth dynamics of strains became evident (Supplementary Figures 1–3). Growth inhibition during 72 h of incubation was fully registered with MIC of EOCs in almost all tests, however, some exceptions were detected (Supplementary Figures 1–3). On the other hand, we observed synergy between EOCs and HLE or EDTA by using sub-MICs of these antimicrobials at 0.25 MIC or 0.5 MIC showing in most cases the same inhibitory effect depending on the strain tested and the combination used (Supplementary Figures 1–3). Furthermore, we observed a short lag phase (1–2 h) in the absence of the treatment (controls), while it was extended in the presence of antimicrobials (Supplementary Figures 1–3).

## Effect of antimicrobials on biofilms of pathogenic multiresistant bacteria

### Biofilm formation capacity of pathogenic multiresistant bacteria

**FIGURE 1:**
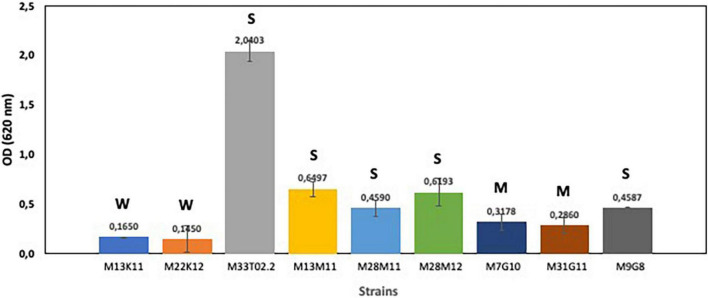
Biofilm formation capacity of *Enterococcus* sp. strains M13M11, M28M11 and M28M12; *Staphylococcus* sp. strains M7G10, M9G8 and M31G11; and *Pseudomonas* sp. strains M13K11, M22K12 and M33T02.2. Data were expressed as mean value (± standard deviations). The cut-off (ODC) was defined as the mean OD value of the negative control. Based on the OD, strains were classified as non-biofilm “N” producers (OD ≤ OD_*C*_), weak “W” (OD_*C*_ < OD ≤ 2 × ODC), moderate “M” (2 × OD_*C*_ < OD ≤ 4 × OD_*C*_) or strong “S” biofilm producers (4 × OD_*C*_ < OD) according to Borges et al. (2012).

First, we analyzed the capacity of enterococci, pseudomonads and staphylococci to produce biofilms. Obtained results showed that all selected strains (*Enterococcus* sp. strains M13M11, M28M11 and M28M12; *Staphylococcus* sp. strains M7G10, M31G11 and M9G8; and *Pseudomonas* sp. strains M13K11, M22K12 and M33T02.2) were biofilm producers in TSB, with this capacity species and strain dependent (Figure 1). On the basis of the OD measurements (at 620 nm), all *Enterococcus* sp. strains were strong biofilm producers. This was also the case for *Staphylococcus* sp. strain M9G8 and *Pseudomonas* sp. strain M33T02.2, with this latter strain being the strongest biofilm former among all the studied strains (Figure 1). However, the values of OD_620_ for the rest of strains were under 1, two *Staphylococcus* sp. strains were moderate biofilm producers while two *Pseudomonas* sp. M22K12 and M13K11 strains showed weak biofilm formation capacity (Figure 1).

### Antimicrobial effect of EOCs, HLE and EDTA on the development of biofilms of pathogenic multiresistant bacteria

**FIGURE 2:**
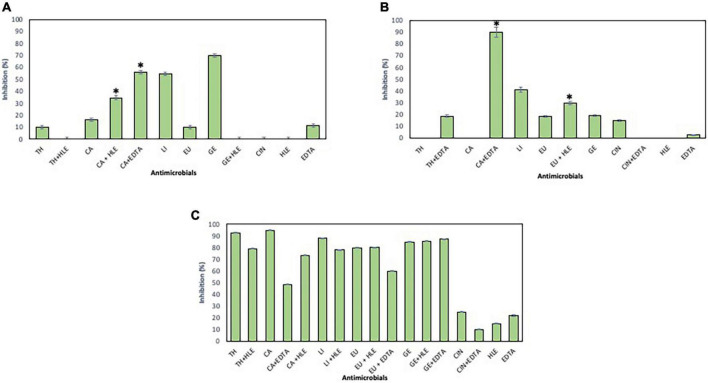
Inhibition of biofilm formation of *Enterococcus* sp. strains M13M11 **(A)**, M28M11 **(B)** and M28M12 **(C)** in the presence of EOCs (TH, CA, LI, EU, GE and CIN), EDTA, HLE or their synergistic combinations at MIC and sub-MIC (0.5 or 0.25) concentrations. Data were expressed as mean value (± standard deviations). *Indicate a statistically significant synergistic effect of antimicrobials.

**FIGURE 3:**
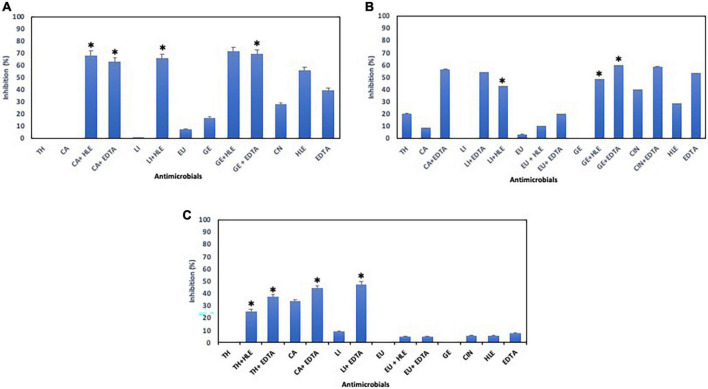
Inhibition of biofilm formation of *Staphylococcus* sp. strains M7G10 **(A)**, M9G8 **(B)** and M31G11 **(C)** in the presence of EOCs (TH, CA, LI, EU, GE and CIN), EDTA, HLE or their synergistic combinations at MIC and sub-MIC (0.5 or 0.25) concentrations. Data were expressed as mean value (± standard deviations). *Indicate a statistically significant synergistic effect of antimicrobials.

**FIGURE 4:**
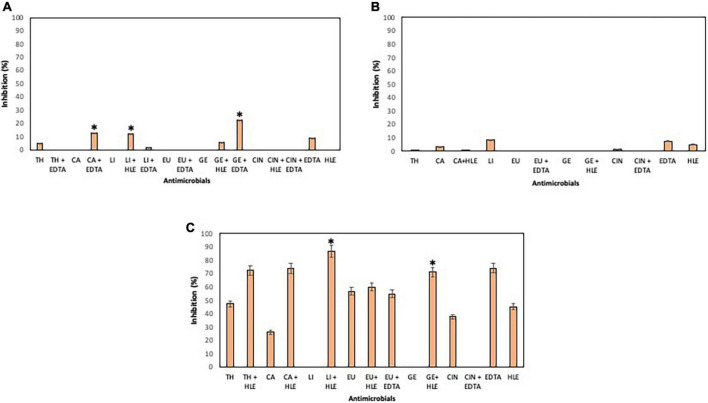
Inhibition of biofilm formation of *Pseudomonas* sp. strains M13K11 **(A)**, M22K12 **(B)** and M33T02.2 **(C)** in the presence of EOCs (TH, CA, LI, EU, GE and CIN), EDTA, HLE or their synergistic combinations at MIC and sub-MIC (0.5 or 0.25) concentrations. Data were expressed as mean value (± standard deviations). *Indicate a statistically significant synergistic effect of antimicrobials.

The inhibitory effect of EOCs, HLE, EDTA and their combinations against developing biofilms of *Enterococcus* sp., *Staphylococcus* sp. and *Pseudomonas* sp. strains was detected depending on the antimicrobial agent and the strain tested (Figures 2–4). Regarding *Enterococcus* sp. strains, EOCs such as CA, LI, GE and EU caused the highest inhibition (> 80%) against *Enterococcus* sp. strain M28M12 (Figure 2C), however, their combination with HLE or EDTA produced no changes or a decreased inhibitory effect (Figure 2C). However, the inhibitory effect of EOCs against the other two *Enterococcus* sp. was less significant with an increased synergistic effect in combination with HLE or EDTA in some cases such as CA or EU (Figures 2A,B). Overall, HLE and EDTA had a small to no effect against enterococci (Figure 2).

With respect to *Staphylococcus* sp. strains, EOCs had a limited inhibitory effect in almost all cases, however, HLE or EDTA exhibited a variable effect (up to 55% in the case of HLE against *Staphylococcus* sp. strain M7G10) (Figure 3). Additive and synergistic effects were detected between EOCs and HLE or EDTA producing an inhibition activity of 50–70% (Figure 3).

On the other hand, developing biofilms of *Pseudomonas* sp. strains were overall less sensitive to EOCs (< 10% of inhibition) (Figure 4) except for *Pseudomonas* sp. strain M33T02.2 to CA, CIN, TH and EU in the range of 26–56% inhibition (Figure 4C). Statistically significant synergistic effects were detected in some cases between CA + EDTA, LI + HLE, GE + HLE, GE + EDTA and TH + HLE (Figure 4).

### Antimicrobial effect of EOCs, HLE and EDTA on preformed biofilms of pathogenic multiresistant bacteria

**FIGURE 5:**
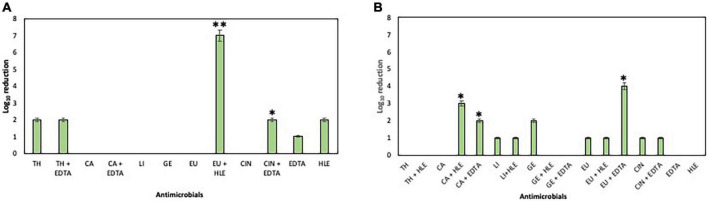
Log_10_ reduction of established biofilms of *Enterococcus* sp. strains M28M11 **(A)** and M28M12 **(B)** after 30 min treatment with EOCs (TH, CA, LI, GE, EU and CIN), EDTA, HLE or their synergistic combinations. (*) and (**) indicate a statistically significant synergistic effect and total elimination of established biofilm, respectively.

**FIGURE 6:**
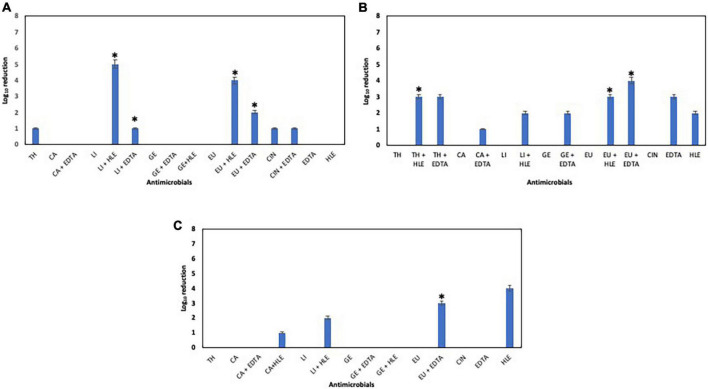
Log_10_ reduction of established biofilms of *Staphylococcus* sp. strains M7G10 **(A)**, M9G8 **(B)** and M31G11 **(C)** after 30 min treatment with EOCs (TH, CA, LI, GE, EU and CIN), EDTA, HLE or their synergistic combinations. *Indicates a statistically significant synergistic effect.

**FIGURE 7:**
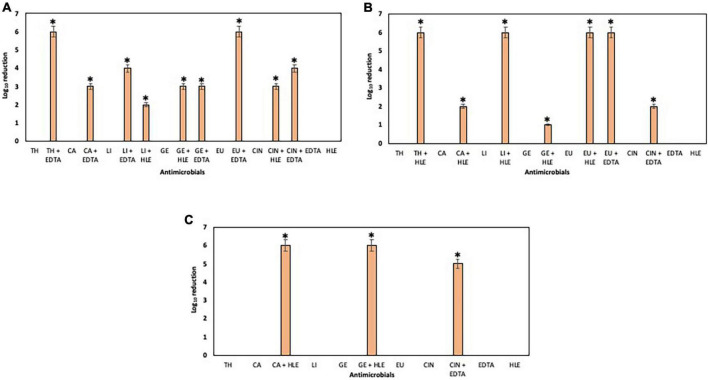
Log_10_ reduction of established biofilms of *Pseudomonas* strains M13K11 **(A)**, M22K12 **(B)** and M33T02.2 **(C)** after 30 min treatment with EOCs (TH, CA, LI, GE, EU and CIN), EDTA, HLE or their synergistic combinations. *Indicates a statistically significant synergistic effect.

The elimination capacity of EOCs, HLE, EDTA or their combinations against established biofilms of enterococci, staphylococci and pseudomonads was dependent on the strain and the antimicrobial used (Figures 5–7). Log_10_ reductions in CFU caused in few cases by some EOCs ranged between 1 and 2 except in the case of *Pseudomonas* sp. strains (no effect). However, it is noteworthy that the synergistic effect of EOCs and HLE or EDTA observed against all strains tested was more pronounced in pseudomonads (Figure 7). On the other hand, HLE and EDTA, in a similar way as EOCs, only inhibited established biofilms of some *Enterococcus* sp. and *Staphylococcus* sp. strains (Figures 5, 6). The synergistic effects detected between EOCs and HLE or EDTA were high in the case of EU + HLE causing complete elimination of established biofilms of *Enterococcus* sp. strain M28M11 (Figure 5), 3 to 4 Log_10_ reductions in CFU of *Staphylococcus* sp. strains (Figure 6) and 6 Log_10_ reductions in CFU of *Pseudomonas* sp. strain M33T02.2 (Figure 7B). Other synergies were detected in the following combinations: EU + EDTA (2–6 Log_10_ reductions in CFU), LI + HLE (2–6 Log_10_ reductions in CFU of staphylococci and pseudomonads), LI + EDTA (1–4 Log_10_ reductions in CFU of staphylococci and pseudomonads), CA + HLE (2–6 Log_10_ reductions in CFU of enterococci and pseudomonads), CA + EDTA (2–3 Log_10_ reductions in CFU of enterococci and pseudomonads), TH + HLE (3–6 Log_10_ reductions in CFU of staphylococci and pseudomonads), TH + EDTA (6 Log_10_ reductions in CFU of *Pseudomonas* sp. strain M13K11), GE + HLE (1–6 Log_10_ reductions in CFU of pseudomonads), GE + EDTA (3 Log_10_ reductions in CFU of *Pseudomonas* sp. strain M13K11), CIN + EDTA (2–5 Log_10_ reductions in CFU of pseudomonads) and CIN + HLE (3 Log_10_ reductions in CFU of *Pseudomonas* sp. strain M13K11) (Figures 5–7).

## Discussion

Multidrug-resistant (MDR) bacteria present a severe and ever-growing global threat to public health. MDR bacteria are spread through different environments -including the food chain- and are directly linked to increased morbidity and mortality, healthcare costs and contamination (van Duin and Paterson, 2016; Cave et al., 2021). To a large extend, this is caused by the irrational use and misuse of antimicrobials (biocides and antibiotics) in different settings (clinical therapy, veterinary, agriculture, industry, etc.), which have increased the prevalence of MDR bacteria and their resistance genes, especially those acquired by horizontal gene transfer. The selective pressure exerted by antimicrobials and the biofilm formation capacity of bacteria create a suitable scenario for gene mobilization, recombination and the activation of the SOS system (DNA repair, changes in the genome and gene expression), increasing by thus the emergence of new bacteria with high robustness and diversified resistomes (Couce and Blázquez, 2009; Lavilla Lerma et al., 2013; Schmithausen et al., 2018). In this sense, the World Health Organization’s (WHO) “*One Health”* approach includes as central aim to reduce the menace of these bacteria and their corresponding resistance genes in humans, animals and the environment (Aguirre et al., 2016). In this context, animals are considered the main reservoir of MDR bacteria promoting the spread in the environment, from slaughterhouse throughout the food chain and subsequently humans (Sobsey et al., 2006). Previous studies performed by our group and others showed that the contamination sources in the slaughterhouse (animals, slaughtering and meat chain production processes) are greatly responsible for the increased risks to food safety and consumer health due to MDR bacteria (Lavilla Lerma et al., 2013, 2014a,2014b; Zhu et al., 2013; Campos Calero et al., 2018, 2020).

In the present study, we analyzed the multiresistance profile of *Enterococcus* sp., *Staphylococcus* sp. and *Pseudomonas* sp. strains isolated from various surfaces throughout the meat production chain of a goat and lamb slaughterhouse—from sacrifice to end of production line (Lavilla Lerma et al., 2013). The results obtained indicated that all *Enterococcus* sp., *Staphylococcus* sp. and *Pseudomonas* sp. strains showed multiresistance to at least one agent in three or more antimicrobial classes, however, phenotypic and genotypic resistance profiles may depend on the species and strain tested. Overall, the high prevalence of phenotypic resistance to beta-lactams (ampicillin and cefuroxime) and tetracyclines in *Enterococcus* sp., *Staphylococcus* sp. and *Pseudomonas* sp. strains is probably due to the use of these antimicrobials as veterinary therapeutic agents and promoted by specific resistance genes (intrinsic or acquired beta-lactamases for resistance to beta-lactams and *tet* genes for resistance to tetracycline under selective stress) or non-specific multidrug efflux pumps. Furthermore, it is conceivable that rifampicin resistance could be connected to a spontaneous *rpoB* gene mutation or may originate from co-selection in the presence of fluoroquinolones (i.e., ciprofloxacin), which is frequently used in animals (Didier et al., 2011). Furthermore, ciprofloxacin has been found in ready-to-eat meat products (Chajȩcka-Wierzchowska et al., 2016) as it is also used in the treatment of community-acquired pneumonia and meningitis. In this sense, high resistance to ciprofloxacin was detected (29/30 of tested strains) which is caused by the mutation of the GyrA subunit of DNA gyrase or alteration of efflux pumps. With respect to the other antibiotics, phenotypic resistance was detected against nitrofurantoin, chloramphenicol, imipenem and gentamycin, however, known resistance genes were not always found. This could point toward and highlight the role of efflux pumps in most antibiotic resistances observed in 70–100% of the tested strains, individually or in synergy with specific resistance genes (known and unknown). Our results show that phenotypic and genotypic resistance does not always align. There are several potential explanations for this discrepancy, such as the presence of “silent genes,” which are only expressed under certain conditions. Furthermore, technical challenges could also play a role in the inconclusive phenotypic and genotypic analysis. In this regard, PCR detection of a single gene inside an operon may overlook the absence of other genes that are necessary for phenotypic expression leading to the abovementioned discrepancy (Chajȩcka-Wierzchowska et al., 2016). Finally, the presence of unknown genes in the detected bacterial strains could confer antibiotic resistance to the AMR bacteria or *via* non-specific efflux pumps (Zgurskaya and Nikaido, 2000; Aeschlimann, 2003; Resch et al., 2008).

To cope with ARB and their ARGs in different environments, several strategies have been developed searching for novel and more effective drugs able to eradicate these bacteria both in planktonic and sessile (biofilm) states. However, only a limited number of strategies have proven successful, especially if taking into account that the antimicrobial agent in question should be both sustainable and environmentally friendly. In this sense, as part of the “*One Health*” strategy antimicrobial agents -such as EOCs (Sharma et al., 2016), the HLE disinfectant solution developed by our group (Abriouel et al., 2018) or EDTA investigated in the present study- represent a highly promising alternative to the currently commonly used ammonium quaternary compounds or toxic detergents (e.g., Hegstad et al., 2010; Buffet-Bataillon et al., 2012). To characterize and evaluate these novel antimicrobial strategies, we determined first the MIC of all antimicrobials against ARB *Enterococcus* sp., *Staphylococcus* sp. and *Pseudomonas* sp. strains. The results obtained showed that EOCs -as concentrated natural plant extracts- had different MICs from 10 to 50 μg/mL for CIN followed by TH, CA, LI and GE (100–400 μg/mL) and EU (200–400 μg/mL) depending on the compound and the strain tested. These results are in line with previously published data on reference strains regarding some EOCs (Walsh et al., 2003; Yadav et al., 2015; Miladi et al., 2016). It has been shown previously that EOCs are a good source of bioactive compounds with antioxidative and antimicrobial activity against pathogens including MDR bacteria (Nazzaro et al., 2013; Mo and Os, 2017). The major components of these oils are terpenoids such as the phenols thymol, carvacrol, and geraniol; phenylpropenes such as eugenol; as well as para-cymene and cinnamaldehyde (Chouhan et al., 2017; Kowalczyk et al., 2020). As reported by Man et al. (2019), the most active oils are oregano (CA source), thyme (TH source), lemon (LI source) and lavender against human pathogens (*Staphylococcus aureus*, *Enterococcus faecalis*, *Escherichia coli*, *Klebsiella pneumoniae* and *Pseudomonas aeruginosa*). The strong inhibitory activity of CIN might be ascribed to the high electrophilic properties of the carbonyl group adjacent to the double bond that makes this compound particularly reactive with nucleophiles, such as protein sulfhydryl and amino groups of the microorganism (Neri et al., 2009). On the other hand, the MIC of the HLE disinfectant solution showed a comparably higher variability, with *Enterococcus* sp. strains showing a higher sensitivity, followed by *Staphylococcus* sp. and *Pseudomonas* sp. strains. This variability may depend on the strain tested since Abriouel et al. (2018) demonstrated the successful antimicrobial effect of the HLE disinfectant solution at low concentration (0.15–0.4%) against different Gram positive and Gram negative bacteria. However, the metal chelating agent EDTA showed different results, with pseudomonads the most susceptible bacteria with low MIC (up to 1 mM, except one case) and staphylococci with high MIC (up to 100 mM). The low concentration of HLE that inhibited growth of MDR bacteria is probably due to the synergistic effect of all antimicrobials present in HLE (hydrogen peroxide, Lactic acid and EDTA) (Abriouel et al., 2018).

In a second evaluation step, we looked for synergistic effects between all antimicrobials (1/2 MIC of EOCs, HLE or EDTA) with the aim to reduce the concentration required for growth inhibition and increase its application potential in the food industry. Previous investigations by our group (Lavilla Lerma et al., 2014c) revealed that the addition of a sub-inhibitory concentration of EDTA (3 mM) reduced the MICs of almost all drugs used (antibiotics or biocides) against enterococci. In this sense our current study revealed that (although the synergistic effect was compound and strain dependent) some combinations had consistently good results. Notably 1/2 MIC of CIN + EDTA, GE + HLE, LI + HLE, LI + EDTA, CA + EDTA or GE + EDTA were the combination of EOCs + HLE most effective against *Pseudomonas* sp. strains and the combination of EOCs + EDTA against *Enterococcus* sp. and *Staphylococcus* sp. strains. Furthermore, these data were corroborated over 72 h when dynamic growth was analyzed at both 1/2 MIC and 1/4 MIC of different antimicrobial combinations to detect delayed or enhanced growth in the presence of some antimicrobials (Theophel et al., 2014). Notably, the results furthermore indicated that in some cases growth inhibition was similar at 1/4 MIC for two antimicrobials suggesting their strong synergistic activity. The different synergistic effects obtained may be due to the differences in cell wall architecture of Gram positive and Gram negative bacteria. In addition, the use of EDTA or HLE (containing EDTA as chelating agent) may facilitate the access of some EOCs to their target and enhancing their antimicrobial activity *via* increased permeabilization and toxic effects on the cytoplasmic membrane structure and function (Magi et al., 2015; Chouhan et al., 2017).

To eradicate MDR bacteria, another challenge must be addressed, i.e., bacterial biofilm formation, which is associated with increased resistance to several antimicrobials (Dzianach et al., 2019; Beloin and McDougald, 2021; Rathod et al., 2021). In this sense, selected MDR bacteria of *Enterococcus* sp., *Staphylococcus* sp. and *Pseudomonas* sp. strains were shown to be biofilm producers with different capacity (mostly strong and moderate), thus it is crucial to determine if the synergistic effects of different antimicrobials obtained previously against these bacteria in planktonic state are still effective against biofilms. Hence, we analyzed the effect of antimicrobials and their combinations against developing and established biofilms. In the first case, developing biofilms of *Enterococcus* sp. M28M12 and *Pseudomonas* sp. M33T02.2 strains were sensitive to EOCs (> 80% of inhibition and 26–56% for *Enterococcus* sp. M28M12 and *Pseudomonas* sp. M33T02.2 strains, respectively), while the other enterococci, staphylococci and pseudomonads were less sensitive. On the other hand, as reported by Kavanaugh and Ribbeck (2012), some EOCs were able to eliminate biofilms of *Pseudomonas* sp. and *S. aureus*, thus we can suggest that the interaction of EOCs with their target is highly specific for each strain (hydrophobicity of the oil, outer membrane and peptidoglycan structure and composition of each strain) independently of the species. In this context, to strengthen the antimicrobial activity of EOCs against developing biofilms, the combination of EOCs (CA, LI, GE, EU, CIN or TH) with HLE or EDTA is promising in some cases, notably through the inhibition of biofilm formation of MDR bacteria. It is also noteworthy that some of the synergistic effects produced against enterococci, staphylococci and pseudomonads in planktonic state were reproduced in developing biofilms.

Once biofilms are established (sessile phase), the effect of antimicrobials is less pronounced and/or even inefficient. This is not surprising, considering that bacteria use this tool for their survival and growth, and consequently present a great challenge to industries and to human health (Rather et al., 2021). In the current study, higher reductions in Log_10_ CFU were obtained with EU + HLE (3–7 Log_10_ CFU, obtaining total elimination of 7 Log_10_ CFU of *Enterococcus* sp. strain M28M11 and 6 Log_10_ reductions in CFU of *Pseudomonas* sp. strain M33T02.2) and EU + EDTA (2–6 Log_10_ reductions in CFU) against established biofilms of enterococci, staphylococci and pseudomonads. Similarly, El-Far et al. (2021) reported that eugenol had an inhibitory effect on biofilm formation and established biofilms of methicillin resistant *S. aureus* clinical isolates in Egypt. Moreover, other studies revealed the efficacy of CA and TH to eliminate preformed biofilms of *S. aureus* (Nostro et al., 2007) and *P. fluorescens* KM121 (Myszka et al., 2016), and CA, TH and EU for biofilm elimination and modification of resistance susceptibility of *Salmonella enterica* serovar Typhimurium strains to nalidixic acid (Miladi et al., 2017). Furthermore, the synergistic combinations of EU and HLE/EDTA, TH, CA, GE, LI or CIN + EDTA/HLE caused Log_10_ reductions in biofilms of several strains (1–6 Log_10_ CFU) depending on the species and the combination used—with *Pseudomonas* sp. strains as most susceptible.

Results obtained for formulations based on the combination of EOCs and HLE or EDTA are encouraging, notably taking into consideration their inhibitory effect on pathogenic bacteria in the planktonic state as well as developing and established biofilms. Furthermore, HLE and EDTA decrease the gene expression of multidrug EfrAB, NorE and MexCD efflux pumps as non-specific resistance mechanisms to several antimicrobials (antibiotics and biocides) as reported by Abriouel et al. (2018) and Lavilla Lerma et al. (2014b), respectively. Moreover, El-Far et al. (2021) demonstrated that eugenol treatment decreased the expression of biofilm related genes (*IcaA*, *IcaD* and *SarA*) which are involved in biofilm development of methicillin resistant *S. aureus* clinical isolates in Egypt. In the same context, it was reported that EU inhibited the expression of adhesion genes *eae* and *ler* (Baskaran et al., 2016) and the expression of migration-related genes *fliC, fimA, lpfA*, and *hcpA*, which encode flagellin A, type 1 fimbriae, long polar fimbriae, and hemorrhagic coli pilus (Hu et al., 2018), leading to significant repression of bacterial quorum sensing (Rathinam et al., 2017) in enterohemorrhagic *Escherichia coli*.

Hence, taking into consideration the safety of EOCs, which are approved by the European Union and the United States food and drug administration as food preservatives (21CFR182.20, 21CFR182.60, 21CFR172.515; 29 March 2022), as well as the safe nature of HLE (composed of natural substances) and EDTA included in the proposed formulations, we suggest these formulations as novel strategy to inhibit, limit and avoid the spread of MDR bacteria, their resistance genes, the development of biofilms or their elimination once established. These highly promising results may be exploited both for medical devices and surfaces as well as in a food industry environment.

## Conclusion

The present study demonstrated that EOCs (TH, CA, LI, GE, EU and CIN), HLE and EDTA have antimicrobial activity against MDR enterococci, staphylococci and pseudomonads. These effects notably depended on the antimicrobial compound and the tested strain. In this regard, follow-up studies will also aim at the molecular characterization of bacterial strains isolated from the slaughterhouse, e.g., identifying the serotypes of the isolated strains. This will notably be useful when connecting information from different studies and add an additional layer of information on the strain/antimicrobial compound interaction.

Synergistic effects of EOCs and HLE or EDTA -used at 1/2 MIC- were detected against planktonic and also developing and established biofilms.

Hence, here we propose novel antimicrobial formulations as a promising sustainable alternative to currently used chemical formulations, notably able to decrease the emergence and spread of MDR bacteria and their resistance genes in the food chain and the environment. This can notably result in the decrease of resistomes and pathogenesis in clinical and industrial areas (environmental surfaces and devices) as well as present a crucial and urgently needed sustainable alternative formulation preserving the antibiotic therapeutic action.

## Data availability statement

The original contributions presented in this study are included in the article/Supplementary material, further inquiries can be directed to the corresponding author/s.

## Author contributions

HA and NB were involved in study design. HA, NC, JM, and NB were involved in data analysis and writing the manuscript. NC and JM did the experiments. SC did the statistical analysis. All authors contributed to the manuscript and have approved its submission.
