# Supplementary material for: Antibacterial and antibiofilm effects of essential oil components, EDTA and HLE disinfectant solution on Enterococcus, Pseudomonas and Staphylococcus sp. multiresistant strains isolated along the meat production chain
Source: Front Microbiol. 2022 Oct 10;13:1014169. doi: 10.3389/fmicb.2022.1014169 (PMC9589356; doi:10.3389/fmicb.2022.1014169)
Supplement: Supplementary file 2 [file Presentation_2.PPTX]

## Slide 1
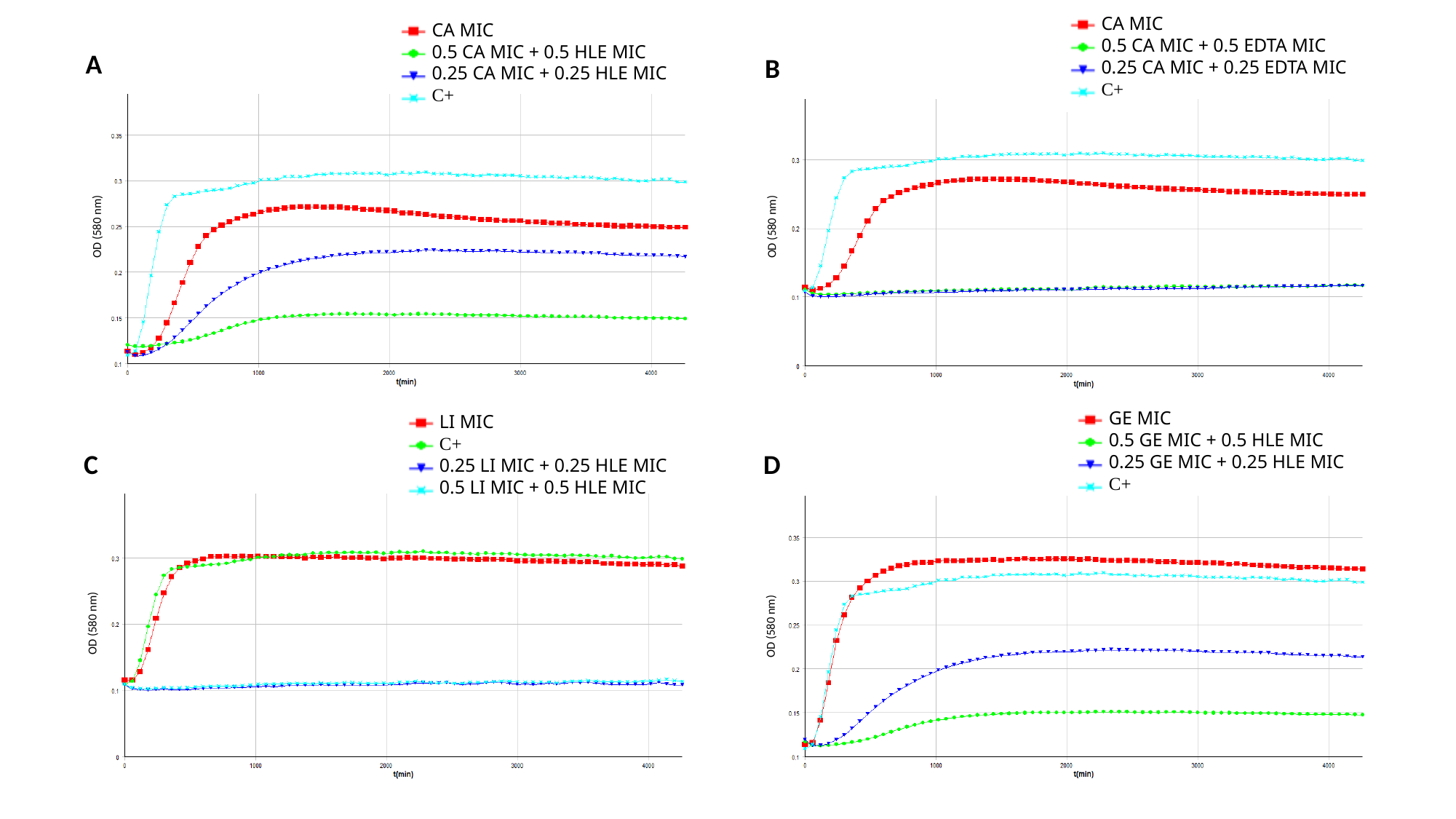

CA MIC
0.5 CA MIC + 0.5 EDTA MIC
0.25 CA MIC + 0.25 EDTA MIC
C+
CA MIC
0.5 CA MIC + 0.5 HLE MIC
0.25 CA MIC + 0.25 HLE MIC
C+
A
B
OD (580 nm)
OD (580 nm)
GE MIC
0.5 GE MIC + 0.5 HLE MIC
0.25 GE MIC + 0.25 HLE MIC
C+
LI MIC
C+
0.25 LI MIC + 0.25 HLE MIC
0.5 LI MIC + 0.5 HLE MIC
D
C
OD (580 nm)
OD (580 nm)

## Slide 2
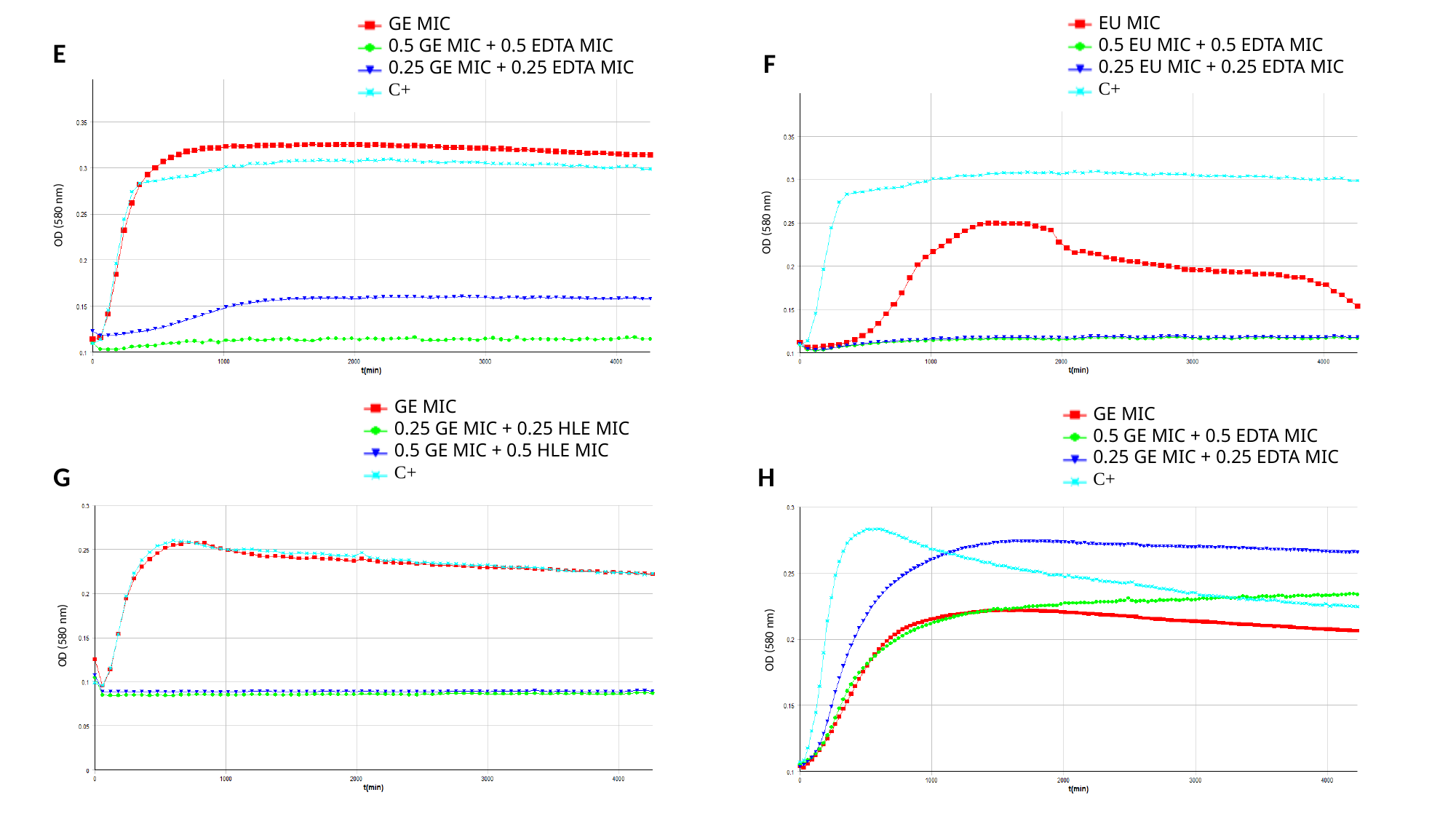

EU MIC
0.5 EU MIC + 0.5 EDTA MIC
0.25 EU MIC + 0.25 EDTA MIC
C+
GE MIC
0.5 GE MIC + 0.5 EDTA MIC
0.25 GE MIC + 0.25 EDTA MIC
C+
E
F
OD (580 nm)
OD (580 nm)
GE MIC
0.25 GE MIC + 0.25 HLE MIC
0.5 GE MIC + 0.5 HLE MIC
C+
GE MIC
0.5 GE MIC + 0.5 EDTA MIC
0.25 GE MIC + 0.25 EDTA MIC
C+
H
G
OD (580 nm)
OD (580 nm)

## Slide 3
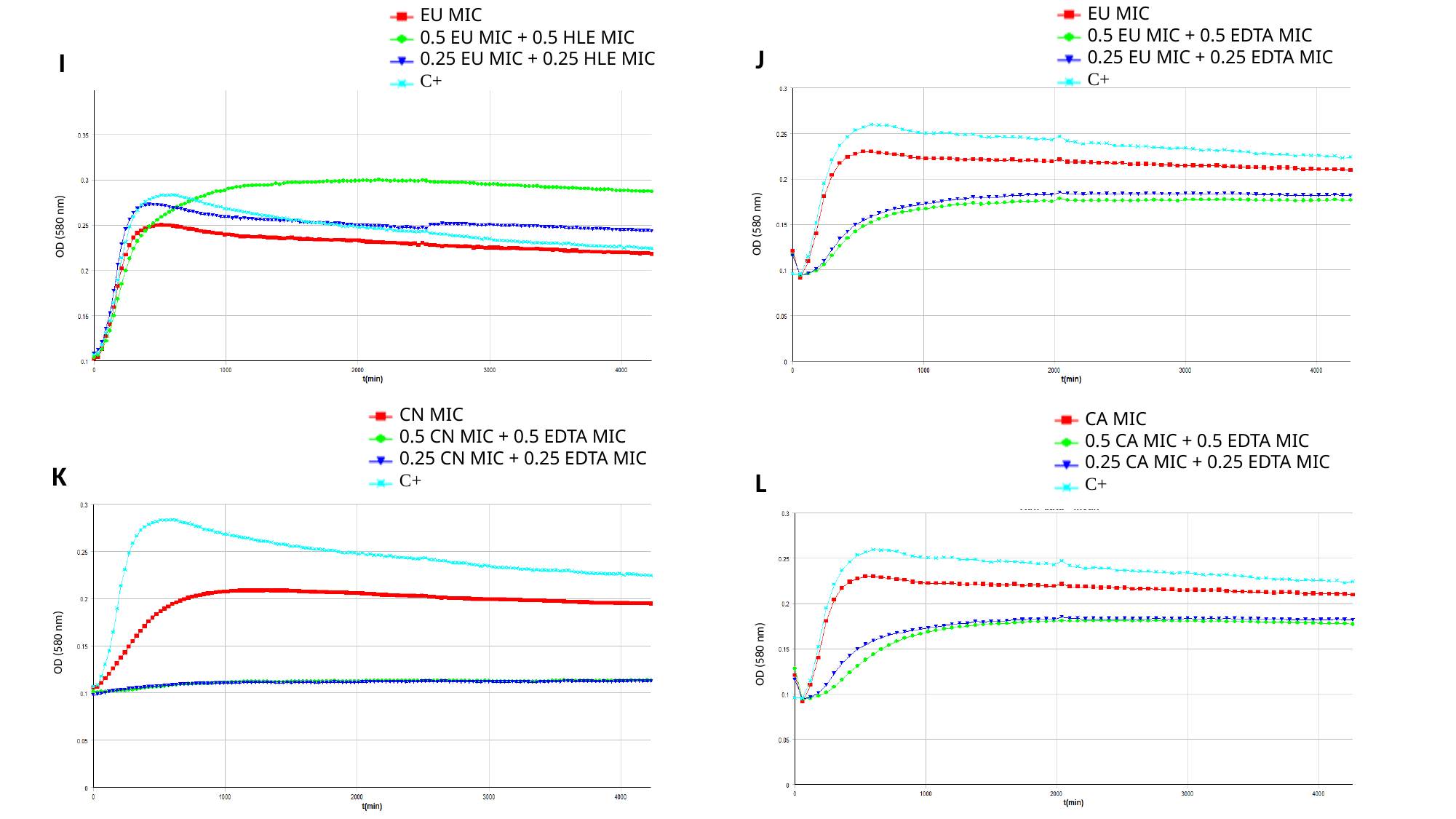

EU MIC
0.5 EU MIC + 0.5 EDTA MIC
0.25 EU MIC + 0.25 EDTA MIC
C+
EU MIC
0.5 EU MIC + 0.5 HLE MIC
0.25 EU MIC + 0.25 HLE MIC
C+
J
I
OD (580 nm)
OD (580 nm)
CN MIC
0.5 CN MIC + 0.5 EDTA MIC
0.25 CN MIC + 0.25 EDTA MIC
C+
CA MIC
0.5 CA MIC + 0.5 EDTA MIC
0.25 CA MIC + 0.25 EDTA MIC
C+
K
L
OD (580 nm)
OD (580 nm)

## Slide 4
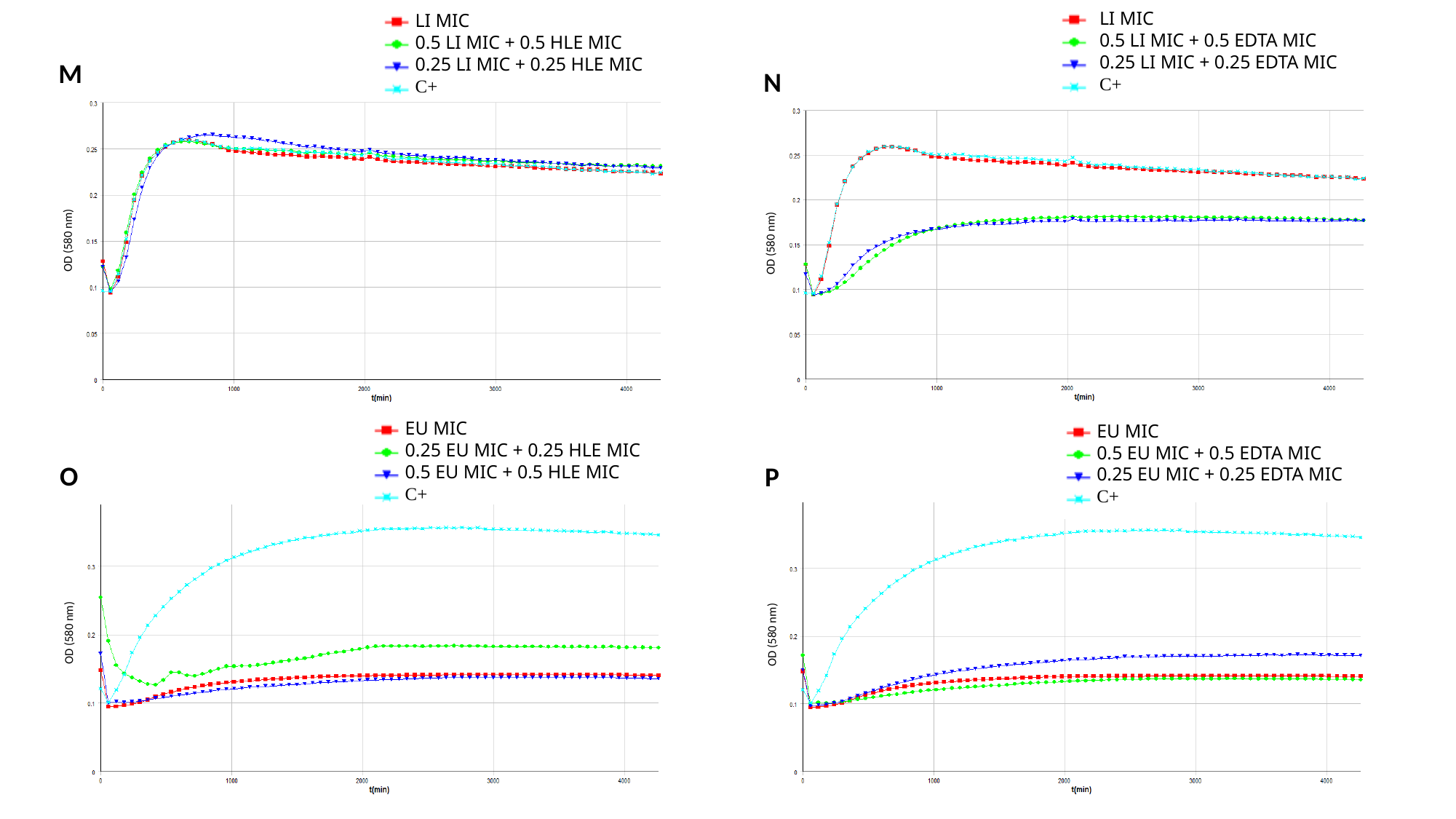

LI MIC
0.5 LI MIC + 0.5 EDTA MIC
0.25 LI MIC + 0.25 EDTA MIC
C+
LI MIC
0.5 LI MIC + 0.5 HLE MIC
0.25 LI MIC + 0.25 HLE MIC
C+
M
N
OD (580 nm)
OD (580 nm)
EU MIC
0.25 EU MIC + 0.25 HLE MIC
0.5 EU MIC + 0.5 HLE MIC
C+
EU MIC
0.5 EU MIC + 0.5 EDTA MIC
0.25 EU MIC + 0.25 EDTA MIC
C+
O
P
OD (580 nm)
OD (580 nm)

## Slide 5
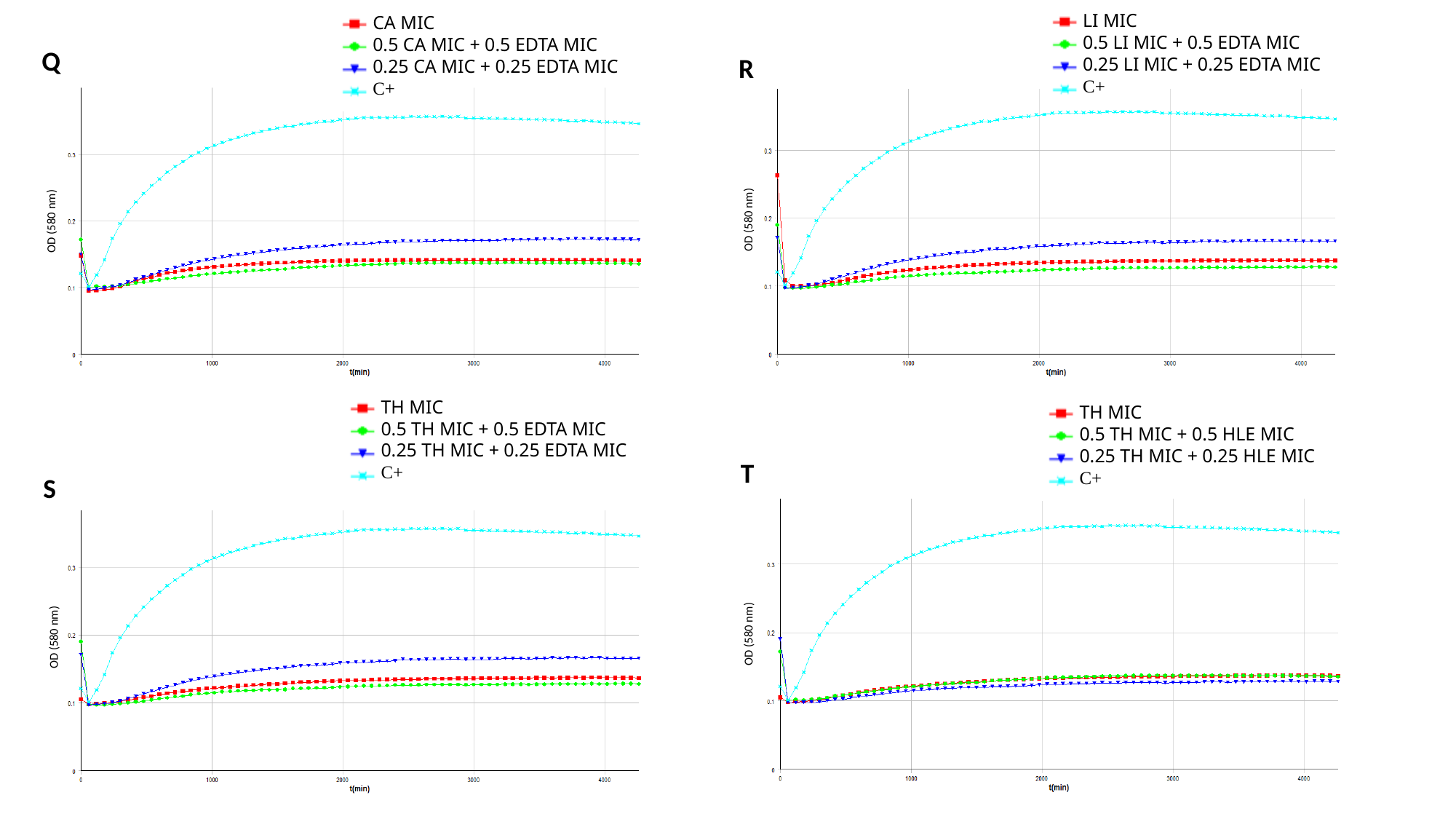

LI MIC
0.5 LI MIC + 0.5 EDTA MIC
0.25 LI MIC + 0.25 EDTA MIC
C+
CA MIC
0.5 CA MIC + 0.5 EDTA MIC
0.25 CA MIC + 0.25 EDTA MIC
C+
Q
R
OD (580 nm)
OD (580 nm)
TH MIC
0.5 TH MIC + 0.5 EDTA MIC
0.25 TH MIC + 0.25 EDTA MIC
C+
TH MIC
0.5 TH MIC + 0.5 HLE MIC
0.25 TH MIC + 0.25 HLE MIC
C+
T
S
OD (580 nm)
OD (580 nm)

## Slide 6
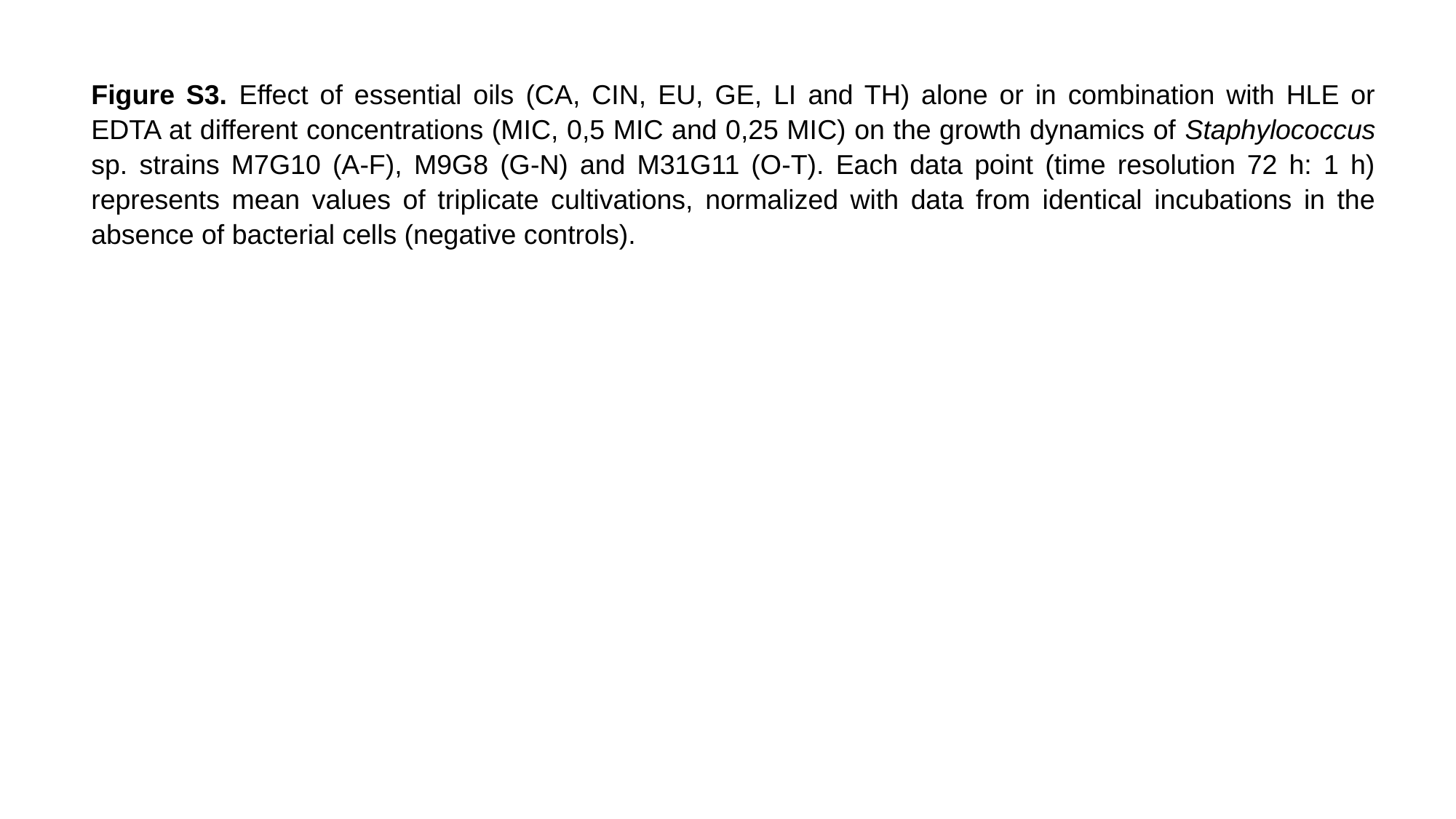

Figure S3. Effect of essential oils (CA, CIN, EU, GE, LI and TH) alone or in combination with HLE or EDTA at different concentrations (MIC, 0,5 MIC and 0,25 MIC) on the growth dynamics of Staphylococcus sp. strains M7G10 (A-F), M9G8 (G-N) and M31G11 (O-T). Each data point (time resolution 72 h: 1 h) represents mean values of triplicate cultivations, normalized with data from identical incubations in the absence of bacterial cells (negative controls).
